# Key Action Areas for Population and Planetary Health: Recommendations Arising From the Transforming the UK Food Systems Programme

**DOI:** 10.1111/nbu.70035

**Published:** 2025-10-15

**Authors:** Emma Hunter, Tracey Duncombe, Alexandra Johnstone, Hannah Mitchell, Roya Shahrokni, Riaz Bhunnoo, Guy Poppy

**Affiliations:** ^1^ The Rowett Institute University of Aberdeen Aberdeen UK; ^2^ Department of Food and Nutritional Sciences University of Reading Reading UK; ^3^ UKRI Global Food Security Programme Swindon UK; ^4^ University of Bristol Bristol UK

**Keywords:** climate change, environment, food system, obesity, population health, recommendations

## Abstract

The UK food system faces critical challenges at the intersection of public health, environmental sustainability, and economic resilience. Currently contributing significantly to greenhouse gas emissions, biodiversity loss, and freshwater use, the system also fails to ensure universal access to healthy diets—particularly for lower‐income populations during a cost‐of‐living crisis. The Transforming UK Food Systems (TUKFS) Programme has brought together academia, industry, policymakers, and communities to co‐produce solutions to these complex challenges. This paper highlights findings from the Programme, with a focus on improving nutrition and sustainability. It outlines four key action areas: (1) innovation in manufacturing and supply chains, including development of UK‐grown pulses, fortified foods, and aquaculture systems; (2) transforming food environments, such as school meals and supermarkets, to make healthy food more accessible; (3) empowering communities through co‐production and stakeholder engagement across the food system; and (4) reforming policy and governance by aligning national and local strategies and applying systems thinking to food policy. Collectively, these actions aim to drive coordinated, evidence‐based transformation toward a healthier, more equitable, and sustainable UK food system.

## 1 Introduction

What we eat is shaped by, and simultaneously shapes, the world in which we live. It is estimated that food system activities account for a third of all greenhouse gas emissions, 70% of freshwater use and a third of biodiversity loss (Crippa et al. 2021; United Nations 2023a). Meanwhile, around three billion people cannot afford to purchase and consume a healthy diet (United Nations 2023a), and poor diet is estimated to be a cause of 22% of adult deaths globally, particularly due to increasing levels of non‐communicable diseases, including cardiovascular disease and type 2 diabetes (Afshin et al. 2019). In 2015, UN Sustainability Goals were introduced to align governments and mitigate urgent global environmental, political and economic challenges (United Nations 2015). The goals centre around ending hunger, promoting wellbeing, reducing the threat of climate change and managing natural resources. However, the current food system, driven by economic and political interests, is not moving to meet these goals (Béné 2022; United Nations 2023b).

The Transforming UK Food Systems (TUKFS) Programme was established in 2020 to provide and assess evidence to tackle food system challenges through a UK lens, supported through a major investment (£47.5 million) by UKRI's Strategic Priorities Fund. With an ethos of co‐production across 16 research projects and a Centre for Doctoral Training, the Programme involves 37 UK academic and research institutions and over 200 organisations from farmers and agricultural organisations, major food retailers and food industry bodies, national and local government actors, as well as charities and consumer groups. Research undertaken within the programme is addressing questions related to what we should eat, produce, manufacture, and import, while considering the complex interactions between health, the environment, and economic factors. These endeavours have generated a large body of data and evidence, much of which has been drawn together in a special edition of the *Philosophical Transactions of the Royal Society B* (Poppy et al. 2025). Comprising 12 original research papers describing the results of projects supported by TUKFS, this volume provides an integrated view of the UK food system and presents practical, workable solutions to re‐orientate the system to promote long‐term, sustainable food‐system transformation. In this news and views article, we highlight some of these findings from a nutrition perspective.

## 2 The Challenge of Transforming UK Diets During an Ongoing Cost of Living Crisis and Climate Emergency

Good nutrition is essential for optimal health and wellbeing throughout the life course (Rodríguez‐Mañas et al. 2023). Enabling citizens to eat in line with food‐based dietary guidelines (FBDGs) (Bechthold et al. 2018), such as the UK Eatwell Guide (GOV.UK 2016), would play a role in reducing obesity prevalence and incur both population‐level health and economic benefits. Additionally, research has shown that eating in line with FBDGs would also benefit the environment (Scheelbeek et al. 2020). However, research suggests only 0.078% of the UK population met all nine Eatwell Guide recommendations (Scheelbeek et al. 2020).

In recent years, adverse weather events, war, and the global COVID pandemic have resulted in disruption to the international food system and a period of rapid food price inflation in the UK (UK Parliament 2024a). Coupled with increases in other commodities such as fuel, together with wage stagnation, this resulted in a cost‐of‐living crisis across the UK (Institute for Government 2022; WWF 2023). The number of UK households reporting food insecurity, defined as ‘limited or uncertain availability of nutritionally adequate and safe foods, or limited or uncertain ability to acquire acceptable foods in socially acceptable ways’ (GOV.UK 2024a), rose to 7.2 million in 2022/23, an increase of 2.5 million from 2021/22 (UK Parliament 2024b). It is estimated that those in the most deprived fifth of the UK population would need to spend 45% of their disposable income to eat in line with the Eatwell Guide, compared to a spend of 11% required by those in the most affluent quintile (The Food Foundation 2025). Healthy food including fresh fruit, vegetables, pulses, and meat contribute to a more expensive shopping basket, compared to less healthy, energy‐dense foods (Dhurandhar 2016; Drewnowski 2009; Hoenink et al. 2024). TUKFS research has shown that people living on a low income are generally aware of what constitutes a healthy diet, but such purchasing and consumption patterns are often inaccessible, unaffordable, and unachievable (Hunter, Stone, Brown, et al. 2025). Sacrificing the healthiness of food purchased and consumed can have implications for body weight, and food insecurity is associated with an increased risk of obesity (Dhurandhar 2016; Drewnowski 2009). Additionally, obesogenic environments, where opportunities to eat healthily may be scarce, encourage weight gain and obesity (GOV.UK 2017).

Obesity, associated with an increased risk of cardiovascular disease, stroke, type 2 diabetes, and some forms of cancer, is a key public health issue in the UK (Scottish Government 2022; GOV.UK 2024b). The estimated annual spend associated with treating obesity‐related diseases of over £6 billion is projected to increase to over £9 billion per year by 2050 (GOV.UK 2017).

Supply chain demands mean farmers and growers are increasingly required to grow crops used for processed foods (cheap raw ingredients), with the need for year‐round availability resulting in fewer crop rotations, less diversity to support wildlife, and subsequent nature loss (Hird 2024). Demands of the current food system also see land that could be used to grow cereals, fruit, and vegetables used to feed animals reared for meat, further fueling climate change (WWF 2022).

Below we present TUKFS evidence across key action areas to promote long‐term, sustainable food‐system transformation to ensure the health of both people and the planet.

## 3 Action Area: Innovating in Manufacturing and the Supply Chain

Innovation throughout the food system is vital to ensure food security in a way that supports the health of a growing population, environmental sustainability, and economic resilience. The TUKFS Programme supports a range of pioneering innovation initiatives that address these multidimensional challenges. This includes research around the use of novel food sources; for example, processing grass, which contains omega‐3 fatty acids, found to improve heart and brain function and reduce inflammation (Mumbi et al. 2024), and which grows in abundance in the UK, into edible food fractions such as oils, proteins, vitamins, and carbohydrates (Harper Adams University 2022). TUKFS research is also exploring opportunities for farmers to diversify their businesses and enable improved agroecological land management, whilst maintaining food production. For example, TUKFS research is exploring modular closed‐loop recycling aquaculture systems (RAS) to grow king prawns on farms. The research demonstrates that RAS units can be powered by renewable energy and require less land and water to produce a high‐quality, healthy source of protein compared to conventional livestock farming (University of Exeter 2025).

Key actions arising from TUKFS research relating to product innovation also include incentivising the production and consumption of UK pulses and the rollout of fortified, biofortified, and healthier genetic variants of bread and other food products. Taking a ‘health by stealth’ approach (FDF 2025; Lovegrove 2024), TUKFS projects explored food reformulation to make it easier for citizens to do just that. Research has examined the reformulation of bakery items to incorporate UK‐grown pulses (Baddeley et al. 2013; Bailes et al. 2018; Stoddard and Schauman 2022) and fortified ingredients (Wilkinson et al. 2025) to improve intake of protein, fibre, vitamins, and minerals and improve population health.

Product innovation offers the opportunity to increase and diversify UK domestic food production at the same time as improving soil health and reducing the impact of livestock overgrazing (Harpers Adams University 2022; University of Exeter 2025). Additionally, this research has the potential to reduce the UK's reliance on imported ingredients such as soya and seafood, where production can be associated with ecosystem destruction. Further, it offers opportunities to increase the resilience of UK supply chains that are susceptible to international disruption and associated price increases as witnessed recently throughout Covid 19, the war in Ukraine, adverse weather events and due to Brexit (Su et al. 2023).

While product and processing innovation can be harnessed to increase the availability of healthy and sustainable foods, there are other pathways that the TUKFS programme has considered to make it easier for people to eat well. The food environment is the space within which food acquisition and consumption occurs (HLPE 2020). Physical, economic, socio‐cultural, and policy conditions influence food choices, access to, and availability of food, as well as food quality and safety (HLPE 2017). Food environments provide the opportunity for intervention and action (Hansen et al. 2022). Rather than focusing on changing population behaviour around eating a healthy diet at the individual level (Adams et al. 2016; Theis and White 2021), the work undertaken within the TUKFS Programme focuses on structural, systems‐level interventions including institutional catering, the supermarket context, and Government policy.

## 4 Action Area: Transforming Food Environments for Healthy and Sustainable Food

Institutional food environments, including those in schools and hospitals, often operate on constrained budgets, which presents a barrier to the procurement of healthy, sustainable food, or a lack of prioritisation of healthy food offerings from leadership, resulting in fluctuations of provision across institutions across the UK (Parnham et al. 2023). Therefore, a key action arising from the TUKFS Programme implores greater focus on changing recipes and menus in hospitality and wider public sector catering to include more sustainably sourced, affordable, and nutritionally dense ingredients. TUKFS Projects have examined the introduction of pulses and underutilised fish species to school canteen menus (King 2025; Sustainable Food Places 2025). In addition to highlighting their acceptability to students, the research points to opportunities to diversify the procurement of institutional food to increase the sustainability and resilience of the supply chain (King 2025; Sustainable Food Places 2025). Further, TUKFS modelling work has explored the reorganisation of institutional canteen dishes on weekly menus to reduce the carbon footprint, as well as the fat, salt, and sugar content of foods on offer (TUKFS 2025). Implementing changes to food environments in this manner causes very little disruption and is likely to be acceptable to citizens (Flynn et al. 2025). Alongside the work undertaken to improve school meal offerings, as part of the TUKFS Programme, routes to increasing free or subsidised school meals, including cost–benefit analysis of different potential government policies, have also been modelled (Osifowora et al. 2025). The results suggest positive returns on investment, including the extension of provision to all pupils from households in receipt of welfare (removing the requirement of households to be earning less than £7400 a year) recently announced by the UK Government (GOV.UK 2025).

Next, we move to consider household food consumption where the majority of food purchases, in high‐income countries like the UK, are from supermarkets (Drewnowski and Rehm 2013; Foreman and Lomas 2021). TUKFS research with stakeholders and partners, including supermarket retailers and people with lived experience, has provided actionable evidence for policymakers around what might help support the purchase of healthy, environmentally sustainable food for people living with complex issues of obesity and food insecurity (Lonnie et al. 2023). One key action arising from this work is the need to scale up supermarket‐based interventions that promote healthier options and reduce the visibility of unhealthy foods (Hunter, Stone, Greatwood, et al. 2025).

The food system is a commercially governed entity—this is acknowledged by the Programme. Accordingly, the interventions highlighted within this article also offer the potential to enhance local and national enterprise and the opportunity for a more robust domestic food chain supply. The interventions all offer benefits for health, the environment, and the economy: consideration of each of these elements is driven by the co‐production ethos embedded within TUKFS research.

## 5 Action Area: Empowering Communities

The UK food system constitutes a complex network, involving a wide range of actors who both shape and are shaped by its dynamics. Achieving feasible and acceptable transformation within such a complex system requires societal problem‐solving that actively engages all relevant stakeholders (Bhunnoo and Poppy 2020; Shaw et al. 2024). Through engagement, communities (i.e., local areas, neighbourhoods, or a group of individuals socially connected in some manner) can be empowered to take action to address the needs of the local area and transform the food system. Co‐production creates an environment where research is conducted with, instead of for, stakeholders. All TUKFS projects demonstrate co‐production through engagement with various stakeholders across the food system, including farmers, industry, retailers, educational institutions, and individuals with lived experience (Shaw et al. 2024; Vickers et al. 2024).

Co‐production is ‘messy’ and complex (Shaw et al. 2024). When done well, it enables stakeholders to work together as equals and learn from each other; however, it can be time intensive and may result in differing perspectives and tensions (Involve n.d.; Shaw et al. 2024). Rather than viewing such tensions as barriers and shying away from such practice, the co‐produced nature of various TUKFS projects provides a means of considering and finding solutions, where possible, to begin to address the often fragmented and siloed facets of the food system. Engaging a diverse range of stakeholders offers the potential to reconcile conflicting views and foster consensus. Therefore, a key action arising from this Programme is the need for co‐production to be the default process for decision making that impacts on communities as well as the need for improved inclusion of food systems actors, from farm to fork, including citizens.

Effective food system transformation relies on strong partnerships between food system actors across different sectors to acknowledge the interdependent relationships between agriculture, processing and manufacturing, distribution, and retail. Partnerships can help to reveal the priorities of different actors within these sectors and allow work to begin to try and align these priorities. Through fostering strong, cross‐sectoral partnerships, a more coordinated, acceptable, and economically viable approach to food system transformation can be adopted. Further, effective research impact hinges on strong partnerships with key stakeholders. Collaborating with stakeholders from the outset ensures research is relevant, addresses real‐world needs, and maximises its potential for real‐world application. This is predicated on building capacity for interdisciplinary and systems research and working effectively with stakeholders across the system, understanding their needs and perspectives, and actively engaging them throughout the research process, from design to dissemination.

Support for early career researchers (ECRs) and postgraduate training has formed a key element of the TUKFS programme's strategy to broaden capacity for partnership working by building the next generation of food systems thinkers. ECRs involved with TUKFS Projects reported opportunities to lead on publications, to engage with interdisciplinary teams and differing methodological approaches, as well as the chance to acquire engagement skills with both public and academic audiences. ECRs also gained firsthand insight into successful academic‐industry relationships, something which has been acknowledged as challenging (Bramley 2019; Milliron et al. 2012; Piernas et al. 2022; Rust et al. 2022). Additionally, ECRs reported receiving guidance on navigating data governance issues, cultivating skills in evidence synthesis and policy communication, gaining essential experience in communication and project management, and also being supported to participate in international conferences, expanding professional networks and strengthening presentation skills, crucial for securing future grant funding and building collaborative partnerships.

## 6 Action Area: Transforming Policy and Governance at Local and National Levels

Coherent food policy is vital for food systems transformation. So, it is crucial to understand how different policy levers interact in order to realise positive change that benefits people and planet and to avoid unintended consequences across food system outcomes and activities (Parsons and Barling 2021). Therefore, another key action arising from the TUKFS Programme is the need to apply a systems approach to policy design. There is a call for the establishment of a formal mechanism to involve non‐government stakeholders, including citizens, in the development of policymaking related to food systems. Such a participatory approach could go some way to alleviating often stigmatising government policies related to social security, accessing and receiving emergency food aid (i.e., using a food bank) or public health campaigns to try and improve diet or tackle obesity prevalence (Hunter, Stone, Brown, et al. 2025; Pettinger and Ellwood 2023). More inclusive ways of working that help to align local and national government policies are also needed.

While many TUKFS research projects have focussed on developing and assessing evidence on place‐based approaches to transformation, this evidence also has the potential to feed into and drive national‐ (and devolved administration‐) level food policy. Local Food Partnerships (LFPs), which bring together citizens, community groups, third sector organisations, businesses, local authorities, and academic institutions to shape and inform strategic approaches to local food systems, are part of a national network fostering innovation in the food system to address public health and environmental sustainability challenges (Parente n.d.). Through knowledge exchange, providing credibility to the food systems work being undertaken at the local level and creating effective links between local level work and national policy, LFPs play a critical role integrating place‐based approaches to wider food system transformation in the UK (Parente n.d.). Accordingly, the need to deliver more effective policy through improving links between national and local actions is a key action recommended by the TUKFS Programme.

## 7 What Next for UK Food System Transformation?

The current food system poses significant risks to both population and planetary health. Urgent and coordinated transformation is required. This article summarises some of the recommended key action areas for future food systems transformation stemming from TUKFS research conducted to date (see Poppy et al. 2025 for a comprehensive overview):

- To support innovation in manufacturing and the supply chain, action is recommended to incentivise the production and consumption of UK pulses as well as to support the rollout of fortified, biofortified and healthier genetic variants of bread and other food products.
- To transform food environments for healthy and sustainable food, action is needed to change recipes and menus in hospitality and wider public sector catering to include more sustainably sourced, affordable and nutritionally dense ingredients. Further, action is recommended to scale up supermarket‐based interventions that promote healthier options and reduce the visibility of unhealthy foods.
- To empower communities, evidence from across the Programme highlights the benefits of co‐production as the default process for decision making that impacts on communities. More broadly, action is needed to improve inclusion of food system actors, from farm to fork, including citizens.
- Lastly, to transform food policy and governance at national and local levels, the evidence calls for action to apply a systems approach to policy design and to deliver more effective policy by improving links between national and local actions.

While further insights from the TUKFS Programme are anticipated, there are currently significant windows of opportunity, for example via the UK Government 10‐year health plan and the new UK Food Strategy, where key action areas identified through the TUKFS programme should be considered. The UK food system is a vital component of national security and a significant contributor to the UK economy. We urge decision makers to use the evidence presented to transform the food system for population and planetary health.

## Author Contributions

**Emma Hunter:** writing – original draft, review and editing. **Alexandra Johnstone:** funding acquisition, writing – review and editing. **Hannah Mitchell:** conceptualisation, writing – review and editing. **Roya Shahrokni:** conceptualisation, review and editing. **Tracey Duncombe:** conceptualisation, writing – review and editing. **Riaz Bhunnoo:** conceptualisation, review and editing. **Guy Poppy:** conceptualisation, review and editing.

## Conflicts of Interest

E.H., H.M., R.S., T.D., and R.B. report no conflicts of interest. A.J. holds voluntary committee roles with The Nutrition Society, British Nutrition Foundation, and Association for Nutrition and currently holds a consultancy to provide nutrition information for a catering company. G.P. is a former BBSRC Executive Chair and Director of the TUKFS programme.

## Data Availability

Data sharing not applicable to this article as no datasets were generated or analyzed during the current study.
